# 亲水柱分离-超高效液相色谱-串联质谱法测定食品中3种色胺类精神活性物质

**DOI:** 10.3724/SP.J.1123.2025.08022

**Published:** 2026-09-08

**Authors:** Xiaolei LIAO, Qin LIN, Yue MEI, Hongshun DOU, Hui XU, Yun HE, Cheng PAN, Fengling JIANG

**Affiliations:** 1. 福建省产品质量检验研究院，国家加工食品质量检验检测中心（福州），福建 福州 350002; 1. Fujian Inspection and Research Institute for Product Quality，National Quality Supervision and Testing Center for Processed Food，Fuzhou 350002，China; 2. 南平市公安局延平分局，福建 南平 353000; 2. Yanping Sub-bureau of Nanping Municipal Public Security Bureau，Nanping 353000，China

**Keywords:** 超高效液相色谱-串联质谱, 食品, 赛洛新, 蟾毒色胺, 赛洛西宾, 固相萃取, ultra performance liquid chromatography-tandem mass spectrometry （UPLC-MS/MS）, foods, psilocin, bufotenine, psilocybin, solid phase extraction

## Abstract

本文建立了亲水柱分离结合超高效液相色谱-串联质谱（UPLC-MS/MS）同时测定食品基质中3种色胺类物质的方法。食品样品经提取、离心，强阳离子固相萃取柱（PCX）净化后，采用Capcell PAK ST（S-5）亲水色谱柱分离，在正离子、多反应监测（MRM）模式下采集数据，稳定同位素内标法定量。方法学研究表明，赛洛新、蟾毒色胺和赛洛西宾在0.025~10.0 ng/mL范围内均呈良好的线性关系，相关系数（*R*^2^）均大于0.995，检出限为0.4~0.6 µg/kg，定量限为1.4~1.9 µg/kg；在低、中、高3个加标水平下，3种色胺类物质的加标回收率为94.3%~113.7%，精密度RSD（*n*=6）为0.4%~4.9%。将该方法应用于糖果、饮料、酒、蜜饯、咖啡、保健食品、调味品、熟肉制品、香辛料和火锅底料等共100份样品的检测，发现共有14份样品检出了蟾毒色胺。经过原料溯源调查，推测阳性样品中蟾毒色胺可能来源于配料中的花椒类香辛料。采集并检测了12份花椒和藤椒样品，均检出蟾毒色胺，含量为23.8~885.0 mg/kg，且花椒样品的子离子扫描质谱图与蟾毒色胺标准品的一致。综上，本文建立的方法适用于食品基质中赛洛新、蟾毒色胺和赛洛西宾的大批量、快速、准确检测，同时发现花椒和藤椒中存在蟾毒色胺本底，为后期食品安全风险评估、非法添加检测、食品开发等相关研究提供参考依据。

## Abstract (en)

The illegal addition of substances in ordinary food poses a serious threat to food safety. Therefore， the detection and identification of such additives have long been important research areas in the field of food safety. Especially， illegal additives are showing new trends now， such as emerging functional varieties， new structural derivatives， and clandestine addition practices. Meanwhile， along with the development of online shopping and logistics， new threats pose a substantial challenge to social governance and food safety. In recent years， there are frequent cases of people consuming wild mushrooms containing hallucinogenic tryptamine toxins out of curiosity， and the abuse of tryptamine psychoactive substances also occurs from time to time. Therefore， the development of a robust and sensitive analytical method is necessary for accurately identifying the risks associated with tryptamine psychoactive substances.To address this issue， a novel method based on ultra performance liquid chromatography-tandem mass spectrometry （UPLC-MS/MS） combined with an isotopic internal standard and solid-phase extraction purification technique was developed for the simultaneous determination of three tryptamines （psilocin， bufotenine and psilocybin） in ten types of ordinary foods. Several optimization procedures were undertaken， involving the chromatographic conditions， mass spectrometric parameters， and sample extraction and purification protocols. The food samples were extracted and centrifuged， followed by purification using a strong cation exchange solid-phase extraction column. The separation was carried out using a Capcell PAK ST （S-5） hydrophilic interaction chromatographic column. The multi-reaction monitoring （MRM） mode was employed for data acquisition in the positive ion mode， whereas isotopic internal standards were used to quantify the three tryptamines. It was verified that the calibration curves of psilocin， bufotenine， and psilocybin exhibited good linearities （*R*^2^>0.995） within the range of 0.025-10.0 ng/mL. The limits of detection （LODs） for psilocin， bufotenine and psilocybin were determined as 0.4， 0.5， and 0.6 µg/kg， respectively， while the limits of quantification （LOQs） were determined as 1.4， 1.7， and 1.9 µg/kg， respectively. The LODs and LOQs demonstrate that the method has the ability to detect minute quantities of tryptamines. The recoveries of the tryptamines were 94.3%-113.7% under three spiked levels， with relative standard deviations （RSDs） ranging from 0.4% to 4.9% （*n*=6）. The consistent and satisfactory recoveries of tryptamines indicate that this method meets the requirements to accurately quantify the target analytes even in a complex matrix. In summary， this newly developed method is rapid， sensitive， accurate， and compliant with relevant regulatory standards； hence， it is suitable for the determination of psilocin， bufotenine and psilocybin in food matrices. As part of the application of this method， a comprehensive investigation was conducted on ten types of actual food samples， including candies， beverages， wines， succade， coffee， health care products， condiments， cooked meat products， spices and hot pot sauce. As a result， out of 100 samples， 14 samples were found to contain bufotenine. Additionally， one sample had a bufotenine concentration of 7.6 mg/kg. By tracing back， it was discovered that the bufotenine in the positive samples was derived from *Zanthoxylum armatum* DC. or *Zanthoxylum Bungeanum* Maxim. Subsequently， 12 samples， containing *Zanthoxylum armatum* DC. and *Zanthoxylum Bungeanum* Maxim， were collected and processed using the same method， which revealed the bufotenine in all samples with the content ranging from 23.8 to 885.0 mg/kg. At the same time， it was found that the daughter ion scanning mass spectra of the *Zanthoxylum Bungeanum* Maxim sample were almost consistent with those of the bufotenine standard. Based on this， it can be firmly concluded that bufotenine is naturally present in *Zanthoxylum armatum* DC. and *Zanthoxylum Bungeanum* Maxim. In conclusion， the developed method and the discovery of the content of bufotenine in *Zanthoxylum armatum* DC. and *Zanthoxylum Bungeanum* Maxim provide a reference framework for future research in the areas of food safety risk assessment， the screening of illegal additives， and food product development.

色胺类精神活性物质是人类较早使用且具有致幻作用的生物碱之一^［1］^。该类物质与哺乳动物组织内的神经递质5-羟色胺（5-hydroxytrypamine， 5-HT）具有相同吲哚环结构和类似的药理作用，仅需较低的剂量即可使人产生幻觉和情绪障碍^［1-4］^。色胺类精神活性物质存在于某些真菌、植物和动物体内^［5-8］^。目前已发现有150多种“致幻蘑菇”中天然含有我国列管目录中的赛洛新（psilocin， PSC）和赛洛西宾（psilocybin， PSB）^［1，5］^，其中以花斑褶伞菇含量最高^［1，6］^；某些蟾蜍的皮肤^［8］^、小檗皮药材^［9］^中天然含有蟾毒色胺（bufotenine， BUT），该物质同样可引起副交感神经兴奋、色彩幻觉等，在美国、英国和澳大利亚属于违禁物质^［6］^，但目前不在我国列管目录中。除了上述天然存在的色胺类物质外，基于该种吲哚环结构人工化学修饰合成的具有相似或更强致幻效果的色胺类“新精神活性物质”（new psychoactive substance，NPS）数量不断增多，截至2022年被发现种类累计达到53种^［2，10］^，这些物质大部分不在我国列管范围内。其中，部分色胺类致幻物质还具有镇痛、治疗成瘾、改善抑郁和焦虑以及其他精神疾病等潜在药用价值^［11-17］^。然而，《2024年世界毒品报告》显示，除部分国家、地区批准赛洛西宾用于医疗外，国外还存在滥用致幻蘑菇或色胺类精神活性物质的“灰色”市场^［18］^。一些不法分子在利益的驱使下将含精神活性物质成分的动植物或色胺类物质非法添加到食品基质中进行售卖，如我国警方就查获过使用色胺类NPS非法添加至巧克力的不法案件^［19］^，这种非法使用、添加色胺类精神活性物质的行为给社会安全埋下了较大隐患。

为满足对色胺类精神活性物质的监测需求，目前报道的分析方法主要包括免疫分析^［20］^、气相色谱-质谱（GC-MS）^［21］^、高效液相色谱-串联质谱（HPLC-MS/MS）^［6］^、液相色谱-高分辨质谱（LC-HRMS）^［22］^、离子迁移谱^［23］^等。但现有文献报道的方法多以生物检材（头发^［24］^、血液^［25］^、尿液^［26］^等）和干制蘑菇^［27］^为检测基质，鲜有针对种类繁多且成分复杂的加工食品。为了满足食品中非法添加色胺类精神活性物质检测、相关司法鉴定技术开发以及出入境监管快速侦测识别非法添加的需要，建立高效、高分辨、高准确度的食品基质中赛洛新、赛洛西宾、蟾毒色胺的分析方法尤为必要。因此，本研究选取多种常见且成分较为复杂的食品基质，建立了快速定性、定量测定赛洛新、赛洛西宾和蟾毒色胺的UPLC-MS/MS测定方法，为食品的安全监管、风险评估以及相关食品研究提供技术支持。

## 1 实验部分

### 1.1 仪器、试剂与材料

Waters Xevo TQ-XS超高效液相色谱-串联质谱仪（美国Waters公司）；Sartorius BSA 224S分析天平（赛多利斯科学仪器有限公司）；Avanti J-E冷冻高速离心机（美国贝克曼公司）；MS3 Basis 涡旋混匀器（德国IKA公司）；DTC-27J超声波清洗器（鼎泰（湖北）生化科技设备制造有限公司）。PWCX固相萃取柱（弱阳离子交换型，60 mg/3mL）和PCX固相萃取柱（强阳离子交换型，60 mg/3mL）购自博纳艾杰尔科技有限公司；20管固相萃取装置（美国安捷伦公司）。

实验用水为超纯水（Milli-Q IQ 7000去离子水发生器，美国Millipore公司）；甲醇（色谱级）和乙腈（色谱级）购自山东禹王实业有限公司化工分公司；甲酸（优级纯）购自德国Merck公司；盐酸（分析纯）、抗坏血酸（分析纯）、氨水（分析纯）和乙酸铵（优级纯）购自国药集团化学试剂有限公司。

赛洛新、赛洛西宾、蟾毒色胺、蟾毒色胺-D4标准溶液（100 μg/mL）均购于天津阿尔塔科技有限公司；赛洛新-D10（4.929 mg，以100%计）、赛洛西宾-D10（100 μg/mL）均购于公安部第三研究所。

### 1.2 实验条件

#### 1.2.1 标准溶液配制

分别准确移取赛洛新、赛洛西宾和蟾毒色胺标准溶液1 000 μL于10 mL容量瓶中，以50%（体积分数，下同）甲醇水溶液（含0.01 mol/L抗坏血酸）作为稀释液定容，配制成10 μg/mL的混合标准储备溶液。赛洛新-D10标准品用稀释液溶解并定容至50 mL，配制成98.58 μg/mL的赛洛新-D10储备溶液；分别准确移取适量体积的赛洛新-D10储备溶液、赛洛西宾-D10和蟾毒色胺-D4标准溶液于10 mL容量瓶中，用稀释液定容，配制成10 μg/mL的混合内标储备溶液。所有储备液放置于-20 ℃冰箱中储存备用。

移取适量混合标准储备液用稀释液配制成100 ng/mL的混合标准中间溶液；移取适量混合内标储备溶液用稀释液配制成100 ng/mL的混合内标中间液。移取适量混合标准中间溶液，并与10 μL混合内标中间溶液混合，用稀释液定容至2.0 mL，逐级配成系列质量浓度（0.025、0.05、0.10、0.25、0.50、1.00、2.50、5.00、10.0 ng/mL）的标准系列工作溶液。所有标准中间液置于4 ℃冰箱中保存，标准工作溶液现配现用。

#### 1.2.2 样品来源与制备

糖果、饮料、酒、蜜饯、咖啡、保健食品、调味品、熟肉制品、香辛料、火锅底料和花椒、藤椒等商品均采购于超市和电商平台，固体或半固体样品用粉碎机打碎、混匀；液体样品直接取用，样品均放置4 ℃冰箱中保存。

#### 1.2.3 样品前处理

称取1 g（精确到1 mg）样品于25 mL容量瓶中，加入250 μL混合内标中间液，再加入20 mL 0.01 mol/L盐酸水溶液（含0.01 mol/L抗坏血酸），涡旋振荡混匀，超声波振荡提取30 min，并定容到刻度，振摇混匀，倒入50 mL塑料离心管中，10 000 r/min离心3 min。准确移取1.0 mL上清液过PCX固相萃取柱（预先用2 mL甲醇和2 mL 0.1%甲酸水溶液活化），待样液流尽后，用2 mL 0.1%甲酸水溶液和2 mL水依次淋洗，真空抽干，再用2.0 mL氨水-甲醇（5∶95，*V/V*）洗脱于5 mL刻度试管中，加入0.5 mL 0.01 mol/L抗坏血酸溶液，涡旋混匀，于40 ℃下氮气吹至约0.5 mL，用50%甲醇水溶液（含0.1%甲酸）定容至2 mL，涡旋混匀。溶液以0.2 µm 聚四氟乙烯（PTFE）滤膜过滤，待测。

### 1.3 分析条件

#### 1.3.1 色谱条件

色谱柱：Capcell PAK ST （S-5）（150 mm×2.0 mm，日本大曹三耀公司）；流动相：A为20 mmol/L乙酸铵水溶液，B为乙腈；洗脱梯度：0～3.0 min，38%A；3.0～5.0 min，38%A～70%A；5.0～6.0 min，70%A；6.0～6.1 min，70%A～80%A；6.1～7.0 min，80%A；7.0～7.1 min，80%A～38%A；7.1～9.0 min，38%A。柱温：35 ℃；流速：0.30 mL/min；进样量：2 μL。

#### 1.3.2 质谱条件

电离源：电喷雾正离子模式（ESI^+^）；毛细管电压：1.00 kV；离子源温度：150 ℃；脱溶剂气温度：500 ℃；脱溶剂气流量：1 000 L/h；反吹气流量：150 L/h；碰撞气：氩气，流量为0.16 mL/min；检测方式：多反应监测（MRM）模式；特征离子及其他质谱参数见表1。

**表1:** 3种色胺类物质、同位素内标的保留时间及质谱参数

Compound	Retention time/min	Precursor ion （*m/z*）	Product ion （*m/z*）	CV/V	CE/eV
Psilocin （PSC）	3.47	205.2	58.0^*^	20	13
			160.1	20	18
Bufotenine （BUT）	4.07	205.2	58.0^*^	20	13
			160.1	20	18
Psilocybin （PSB）	5.39	285.2	58.0^*^	15	25
			205.2	15	17
PSC-D10	3.49	215.3	66.0^*^	20	13
BUT-D4	4.07	209.4	60.0^*^	20	13
PSB-D10	5.39	295.3	66.0^*^	15	25

CV： cone voltage； CE： collision energy； * quantitative ion.

## 2 结果与讨论

### 2.1 质谱条件优化

在ESI^+^模式下，采用流动注射模式将质量浓度均为1 μg/mL的3种色胺类标准溶液和3种内标溶液分别进行母离子和子离子扫描。结果发现，3种色胺类化合物及其内标物均存在［M+H］^+^的分子离子峰，赛洛新和蟾毒色胺为同分异构体，母离子*m/z*均为205.2，赛洛西宾母离子*m/z*为285.2，通过优化锥孔电压等参数得到各目标化合物母离子最大响应值，再对各个目标化合物二级碎裂离子进行扫描优化，选取响应值较高的2个碎片离子分别作为定量、定性离子，得到优化后的碰撞能量等参数。各目标化合物的定量和定性离子对及内标离子对相应的质谱参数见1.3.2节。

### 2.2 色谱条件优化

#### 2.2.1 色谱柱的选择

赛洛新、赛洛西宾和蟾毒色胺都是强极性物质，在反相色谱中难以保留，且赛洛新和蟾毒色胺互为同分异构体，需在色谱上分离。为此，在优化的流动相体系下，本实验比较了HSS T3（100 mm×2.1 mm，1.8 μm，美国Waters公司）和Capcell PAK ST （S-5）（150 mm×2.0 mm，日本大曹三耀公司）两种亲水作用色谱柱对目标化合物的分离效果。实验发现，由于赛洛西宾是两性离子型生物碱且存在极性较强的磷酸基团，HSS T3柱对赛洛西宾的保留能力较差，赛洛西宾出峰过快难以与基质中其他强极性物质分离，从而导致极性物质对赛洛西宾的离子抑制效应较大；而常用于链霉素等强极性化合物分离的Capcell PAK ST （S-5）柱在本实验中能较好地分离3种色胺类目标物，且可获得较好的色谱峰形和响应值。因此，本实验选择Capcell PAK ST （S-5）色谱柱对这3种色胺类目标化合物进行分离。

#### 2.2.2 流动相的优化

本实验以Capcell PAK ST （S-5）色谱柱作为分析柱，以乙腈为有机相，比较了水、0.1%甲酸水溶液、5 mmol/L乙酸铵水溶液、20 mmol/L乙酸铵水溶液和20 mmol/L乙酸铵水溶液（含0.1%甲酸）5种水相流动相体系对目标化合物色谱峰形和响应值的影响。实验结果表明，水相中加酸降低pH值虽有利于提高赛洛新和蟾毒色胺的响应值，但会降低赛洛西宾的响应值；而提高水相中乙酸铵的含量有利于改善赛洛新、赛洛西宾和蟾毒色胺的色谱峰形。综上所述，发现以20 mmol/L乙酸铵水溶液为流动相时，3种目标化合物的信号响应和峰形的综合效果较好，故选择20 mmol/L乙酸铵水溶液-乙腈为最终流动相。

在此基础上，由于赛洛西宾易与极性杂质共流出从而影响方法灵敏度，本实验优化了20 mmol/L乙酸铵水溶液流动相初始比例从65%~35%的分离效果，最终确定流动相条件见1.3.1节。在最优色谱条件下，3种色胺类标准品的总离子流（TIC）色谱图和3种色胺类标准品及其对应内标物的多反应监测（MRM）色谱图见图1。

**图1:**
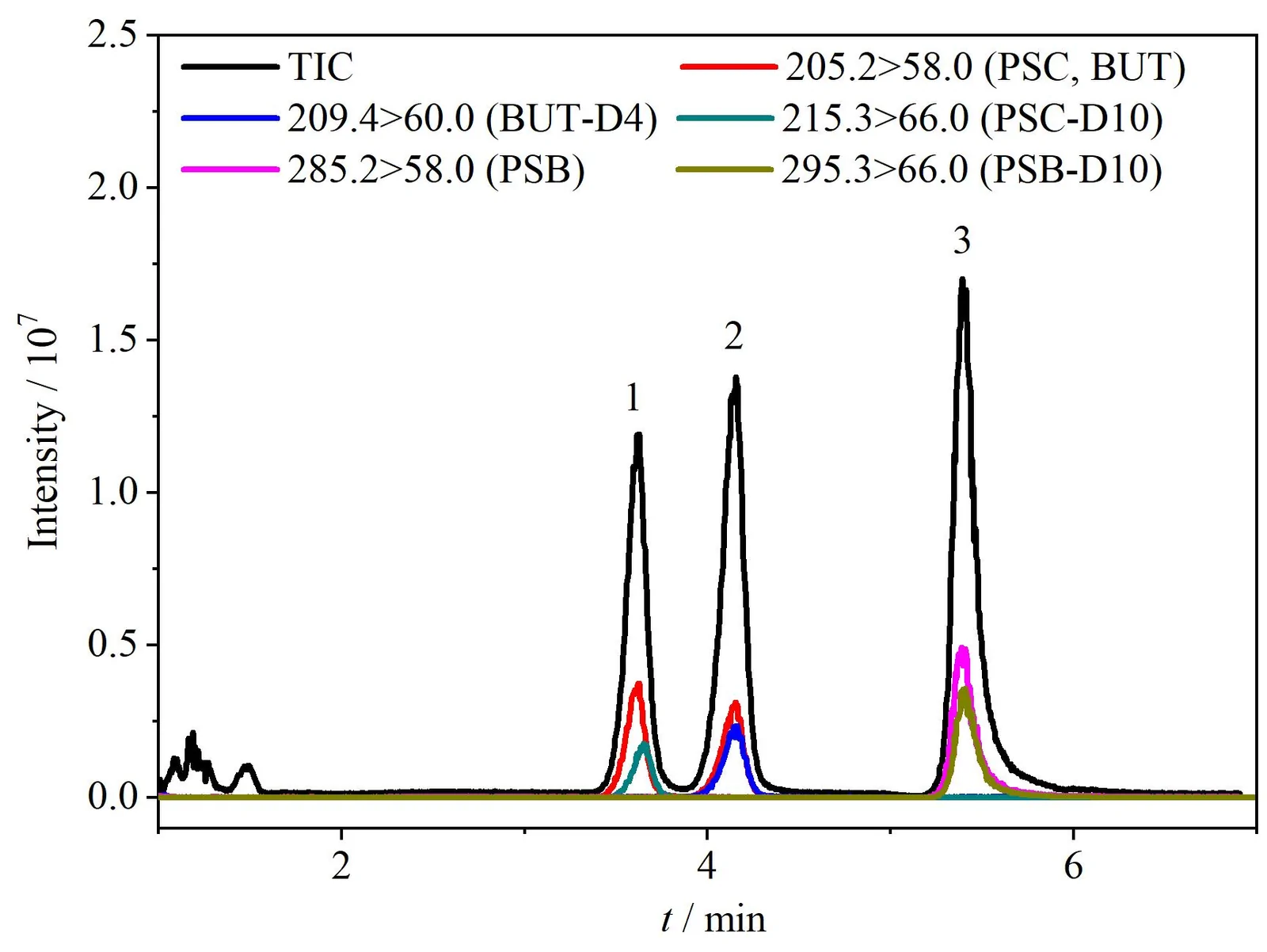
3种色胺类标准品的TIC图和3种色胺类标准品及其对应内标物的MRM图

### 2.3 前处理条件优化

#### 2.3.1 提取溶剂选择

赛洛新、赛洛西宾和蟾毒色胺都是强极性物质，易溶于水。本实验比较了水、乙腈、0.01 mol/L盐酸水溶液、0.01 mol/L盐酸水溶液（含0.01 mol/L抗坏血酸）和0.01 mol/L 盐酸水溶液（含0.01 mol/L抗坏血酸）-甲醇（1∶1，*V/V*）5种提取液对目标化合物的提取效果。实验发现赛洛西宾在乙腈中的提取率较低，可能是因为赛洛西宾含有极性较大的磷酸基团，较难与极性小的乙腈相溶；当以水作为提取溶剂时，发现在中性条件下，赛洛西宾易脱磷酸转变为赛洛新；而在0.01 mol/L盐酸水溶液中，虽能有效抑制赛洛西宾脱磷酸化，提高赛洛西宾的提取率，但由于赛洛新和赛洛西宾不稳定，在空气和水中均易分解；文献［^6^，^29^］报道通过加入抗坏血酸能有效提高赛洛新和赛洛西宾在溶液中的稳定性，为了提高目标物的稳定性和提取率，本实验在提取溶剂中加入适量抗坏血酸，结果显示0.01 mol/L盐酸水溶液（含0.01 mol/L抗坏血酸）对3种色胺类目标物的提取率高于0.01 mol/L盐酸水溶液；而对于0.01 mol/L盐酸水溶液（含0.01 mol/L抗坏血酸）-甲醇（1∶1，*V/V*），因提取溶液中含甲醇，导致强极性的赛洛西宾在固相萃取过程中易被洗脱损失。因此，实验最终选择0.01 mol/L盐酸水溶液（含0.01 mol/L抗坏血酸）为提取溶剂。

#### 2.3.2 固相萃取填料的比较及萃取步骤的优化

本实验选择的食品基质复杂，多数含有大量极性物质。通过查阅文献［^6^］，对于赛洛新和蟾毒色胺的净化采用离子交换型固相萃取柱。考虑到赛洛新、蟾毒色胺在酸性条件下形成阳离子，赛洛西宾含有磷酸基团，故本实验比较了具有阳离子交换和反相保留双重作用的PWCX固相萃取柱和PCX固相萃取柱。实验通过比较目标组分的信号响应及峰形来考察固相萃取柱和净化步骤的净化效果。实验用3种化合物加标水平均为1.0 ng/mL的酱油提取液比较两种净化柱的净化效果，经过相同净化处理后，检测结果表明PCX柱与PWCX柱对赛洛新和蟾毒色胺均能获得较好的信号响应以及峰形，但PCX柱净化后赛洛西宾的信号响应值以及峰形均明显优于经PWCX柱净化的效果，可能是因酱油基质中含有大量阳离子物质易造成赛洛西宾在PWCX柱上的竞争吸附中大量损失（见图2a），故本实验采用PCX固相萃取柱净化食品基质。

**图2:**
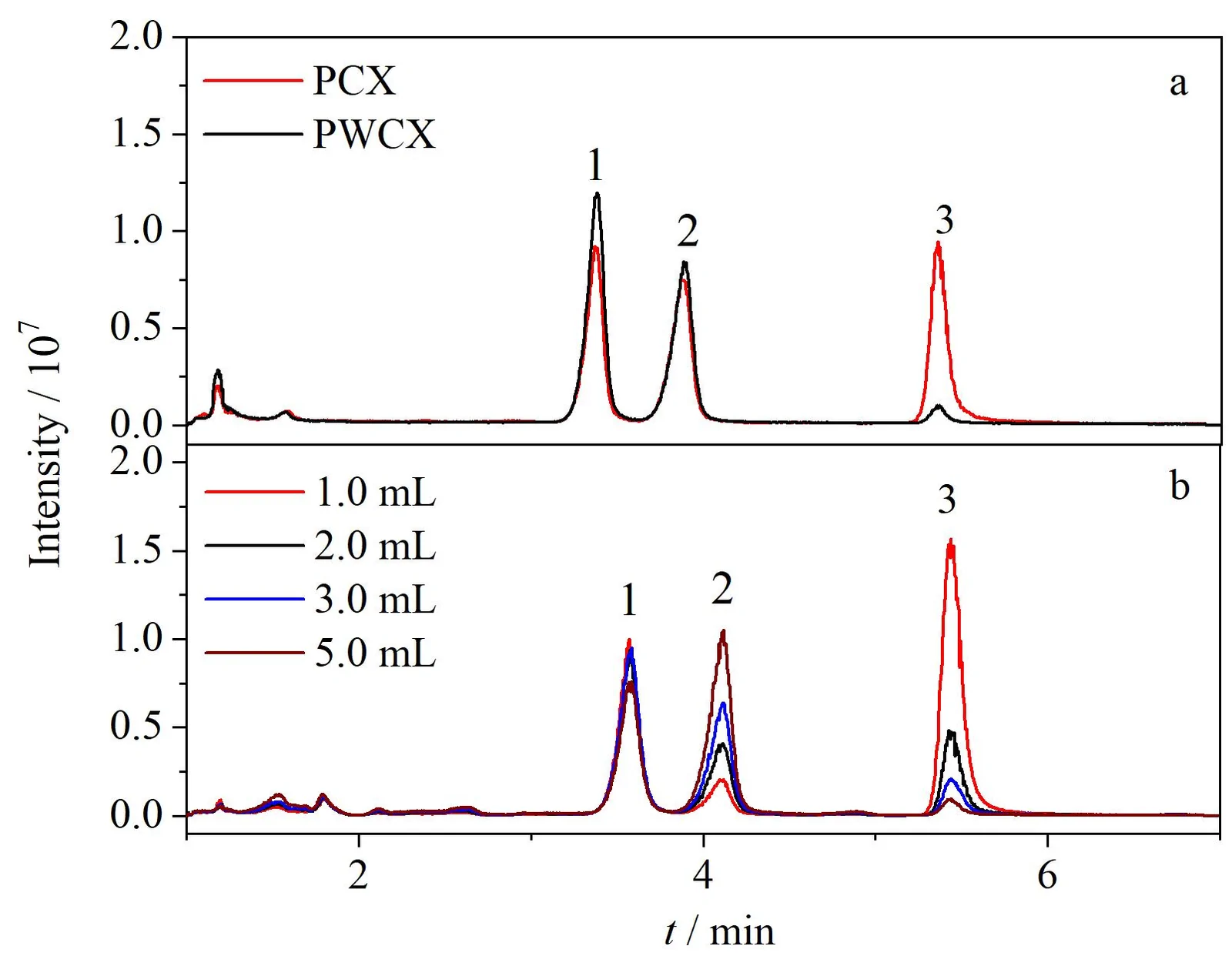
采用不同（a）固相萃取柱类型和（b）上样体积时酱油加标样品（1.0 ng/mL）的TIC图

在对PCX净化柱淋洗液的选择过程中，发现净化柱用2 mL 50%甲醇水溶液淋洗，对赛洛新和蟾毒色胺的回收率无明显影响，但会造成赛洛西宾大量流失，对赛洛西宾的回收率影响较大。因此，实验最终仅采用2 mL 0.1%甲酸水溶液和2 mL水淋洗去除杂质。同时，由于复杂样品基质的洗脱液中仍含有较多杂质，易对强极性的赛洛西宾造成较强的离子抑制。考虑到上样体积与离子抑制效应成正相关，实验比较了1.0、2.0、3.0和5.0 mL酱油加标提取液（1.0 ng/mL）上样净化后的赛洛西宾信号响应值（见图2b），结果发现上样体积越大，赛洛西宾的响应值越小，即提取液的上样体积与赛洛西宾的信号响应值呈负相关。因此，为降低离子抑制效应并提高赛洛西宾检测的灵敏度，实验最终选择1.0 mL为净化的上样体积。

极性较强的色胺类物质用阳离子交换型固相萃取柱净化时，一般使用氨水甲醇溶液进行洗脱，本实验采用氨水-甲醇（5∶95，*V/V*）洗脱PCX柱，并比较了0.5、1.0、1.5、2.0和2.5 mL不同洗脱体积对3种色胺类目标物回收率的影响，结果详见图3。当洗脱液体积为2.0 mL时，3种色胺类物质回收率为97.6%~100.7%；当洗脱液体积为2.5 mL时，目标物回收率与洗脱液体积为2.0 mL时无明显变化，即目标物已全部洗脱。因此，本实验选择2.0 mL作为优化后的洗脱液体积。

**图3:**
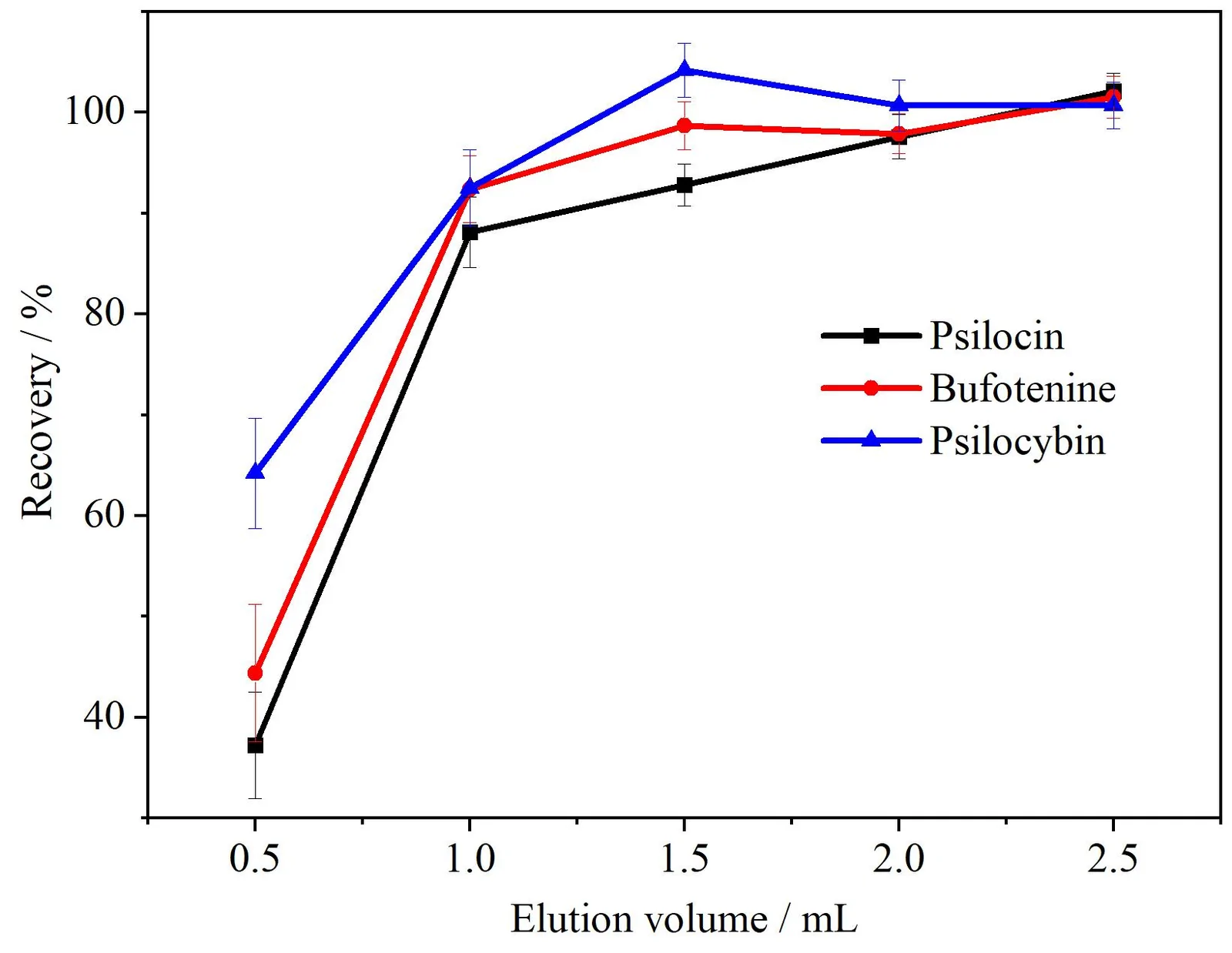
洗脱体积对3种色胺类物质回收率的影响（*n*=3）

### 2.4 基质效应以及进样体积的考察

虽然前处理过程中可将食品基质中大部分杂质净化除去，但残留的杂质仍有可能与目标化合物共同进入离子源，影响目标化合物的离子化效率，这种现象叫基质效应（matrix effect，ME）。本实验参考Chen等^［30］^报道的方法，并选取基质较复杂的蜜饯、酱油、火锅底料和黑胡椒酱作为典型基质，通过测定含有相同浓度目标化合物的基质溶液和纯溶剂标准溶液的响应峰面积，计算并评价基质效应。根据ME=（基质加标溶液峰面积/试剂标准溶液峰面积-1）×100%计算，当ME为正值时，表现为基质增强效应；当ME为负值时，表现为基质抑制效应；其中|ME|>50%为强基质效应，20%≤|ME|≤50%为中等基质效应，|ME|<20%为弱基质效应。本实验考察了目标化合物在0.1 ng/mL加标水平下不同进样体积的基质效应，详见表2。结果表明，在蜜饯、酱油、火锅底料和黑胡椒酱食品基质中3种色胺类物质均呈现出不同程度的抑制效应，并且发现进样体积的变化对蟾毒色胺的基质效应影响相对较弱；但进样体积对赛洛新和赛洛西宾的基质效应影响较大，呈现增强的趋势，尤其在酱油和黑胡椒酱基质中较为明显。因此为降低基质抑制效应对定量结果的影响，本实验采用稳定同位素内标校正，并选择2 µL作为优化后的进样体积。同时发现本实验中蟾毒色胺的基质效应较弱，当样品检出浓度超出线性范围时，可稀释到标准曲线浓度范围内后采用外标法定量。

**表2:** 3种色胺类物质在4种基质中的基质效应

Compound	MEs/% （2 µL/5 µL/10 µL）			
	Succade	Soy sauce	Hot pot sauce	Black pepper sauce
PSC	‒30.3/‒22.1/‒25.9	‒14.7/‒61.7/‒63.8	‒6.5/‒11.7/‒17.5	‒10.0/‒56.3/‒57.7
BUT	‒15.2/‒11.7/‒12.2	‒12.4/‒8.5/‒25.5	‒8.9/‒1.6/‒23.9	‒18.3/‒4.7/‒6.0
PSB	‒22.0/‒18.2/‒38.6	‒76.8/‒96.9/‒99.1	‒1.1/‒6.7/‒13.0	‒71.6/‒94.8/‒97.8

### 2.5 方法学验证

#### 2.5.1 线性范围、检出限和定量限

配制含混合内标质量浓度均为0.5 ng/mL的系列质量浓度（0.025、0.05、0.10、0.25、0.50、1.00、2.50、5.00、10.0 ng/mL）混合标准工作溶液，按1.3节条件进行测定。以标准工作溶液质量浓度为横坐标（*X*），以定量离子峰面积与内标峰面积的比值为纵坐标（*Y*），绘制标准工作曲线，并计算各待测物线性回归方程及其相关系数。如表3所示，赛洛新、蟾毒色胺和赛洛西宾在0.025~10.0 ng/mL范围内具有良好的线性关系，相关系数（*R*^2^）均>0.995。

**表3:** 3种色胺类物质的回归方程、相关系数、检出限和定量限

Compound	Regressionequation	*R*^2^	LOD/（µg/kg）	LOQ/（µg/kg）
PSC	*y*=2.45*x*-0.00066	0.9993	0.4	1.4
BUT	*y*=1.33*x*-0.00230	0.9997	0.5	1.7
PSB	*y*=1.54*x*-0.00290	0.9990	0.6	1.9

*y*： peak area ratio of the three tryptamines to internal standards； *x*： mass concentration， ng/mL.

考虑到酱油基质对目标物基质效应的影响较大，本实验取空白酱油进行低水平（0.05 ng/mL）加标，以信噪比为3倍和10倍时的含量分别作为方法的检出限（LOD）和定量限（LOQ）。如表3所示，赛洛新、蟾毒色胺、赛洛西宾的LOD分别为0.4、0.5和0.6 μg/kg，LOQ分别为1.4、1.7和1.9 μg/kg。

#### 2.5.2 回收率和精密度

本实验在糖果等10种空白食品基质中分别添加低、中、高3个水平（2.50、5.00、50.0 μg/kg）的混合标准工作溶液，各水平制样6份，用于考察方法的准确度和精密度，结果见表4。实验结果显示，该方法对3种色胺类物质的回收率为94.3%～113.7%，精密度为0.4%～4.9%。该方法具有良好的准确度和精密度，适合多类食品样品的检测。

### 2.6 实际样品检测

采用本实验建立的方法对从市场和网络购买的100份样品进行测定。除赛洛新和赛洛西宾均未检出外，共有14份样品检出了蟾毒色胺，阳性样品检测结果见表5。蟾毒色胺检出率为14%，其中最高检出含量为7.6 mg/kg。

**表4:** 不同基质中3种色胺类物质在3个加标水平下的回收率和RSD（*n*=6）

Sample	Added/（µg/kg）	Recoveries/%			RSDs/%		
		PSC	BUT	PSB	PSC	BUT	PSB
Candy	2.50	100.6	105.2	103.6	2.3	2.2	2.9
	5.00	102.1	103.1	100.7	0.9	2.7	2.2
	50.0	98.9	103.5	98.2	1.7	1.8	1.7
Coffee	2.50	98.3	101.5	97.6	1.5	3.2	3.0
	5.00	102.7	99.9	100.3	2.5	1.9	1.5
	50.0	101.8	102.4	103.1	1.9	1.1	1.7
Health supplement oral liquid	2.50	101.5	102.7	99.1	1.3	2.9	1.7
	5.00	101.2	107.2	101.0	1.6	1.9	2.1
	50.0	95.2	105.1	99.1	0.6	1.5	1.0
Chocolate	2.50	102.0	100.9	102.2	1.8	1.9	1.4
	5.00	101.6	105.2	99.6	1.0	0.8	2.0
	50.0	98.3	102.7	97.8	2.4	1.2	1.6
Beverage	2.50	99.8	99.7	100.4	0.9	1.1	1.5
	5.00	102.2	100.6	101.7	0.4	1.5	1.0
	50.0	94.3	103.7	96.4	0.6	1.0	1.1
Wine	2.50	105.5	102.5	100.1	1.1	1.8	1.2
	5.00	103.1	100.8	102.6	1.6	0.9	1.2
	50.0	95.5	105.3	98.5	0.4	1.2	1.8
Succade	2.50	101.2	113.7	101.8	2.3	1.7	4.9
	5.00	105.1	111.9	108.9	2.4	2.9	2.1
	50.0	113.4	112.1	105.1	1.8	1.5	2.6
Soy sauce	2.50	100.3	100.2	98.8	4.5	1.2	3.3
	5.00	100.2	98.5	97.3	1.9	1.9	2.6
	50.0	109.7	108.7	98.6	0.7	2.1	1.7
Sauced meat products	2.50	104.9	103.2	102.7	3.0	3.2	2.2
	5.00	108.9	105.1	101.3	2.2	2.5	3.1
	50.0	100.2	108.9	99.6	2.6	1.9	2.5
Hot pot sauce	2.50	103.8	103.3	99.5	2.1	3.0	2.8
	5.00	99.7	101.5	102.7	2.2	2.4	1.8
	50.0	98.4	107.4	100.5	1.8	1.9	1.1

**表5:** 阳性样品中蟾毒色胺检测结果（*n*=3）

Sample	Bufotenine content/（mg/kg）
Spiced salt	5.7
Spicy hot seasoning	1.7
Oil chili pepper seasoning	0.085
Barbecue seasoning powder	0.019
Garlic-flavored chili seasoning	0.21
Spicy hot seasoning	7.6
Butter hotpot seasoning	0.095
Spicy hot seasoning	0.60
Spicy smoked sausage	0.036
Black pepper sauce	0.0030
Marinated chicken wings	0.0037
Fried and marinated potato strips	0.0019
Marinated small fish	0.045
Spicy sausage	0.48

蟾毒色胺主要存在于蟾酥、特定蘑菇和小檗皮等天然动植物以及真菌中^［9，22，25］^，在加工食品中较少被检出。2024年文献［^28^］报道在自制调味酱中检出蟾毒色胺，但未对该调味酱中蟾毒色胺进行溯源。本实验对上述14份阳性样品标示的配料进行对比分析，发现配料中均含香辛料且多数含有花椒，采用本实验建立的方法对辣椒、桂皮、八角和花椒等香辛料进行测定，发现花椒中含有较高含量的蟾毒色胺，这在以往花椒天然组分研究^［31］^中鲜有报道。为验证该物质确为蟾毒色胺，本实验在子离子扫描（Daughter Scan）模式下，将花椒样品按1.2.3节处理后，经色谱柱分离，对获得的疑似蟾毒色胺的色谱峰进行扫描，并将*m/z*为205.2的母离子对应的子离子的碎片质谱图与同条件下蟾毒色胺标准品扫描获得的子离子的碎片质谱图进行比较（详见图4），结果显示两个谱图基本吻合。为进一步确证花椒中存在蟾毒色胺本底以及探究本底含量情况，网购不同产地的青花椒、红花椒和藤椒等花椒属植物果实共12份样品进行测试，均检出蟾毒色胺，含量为23.8~885.0 mg/kg（详见表6），且测定结果显示鲜花椒中蟾毒色胺含量明显高于干花椒。花椒和藤椒被广泛用作天然调味品，且在我国具有较长的食用历史，虽然花椒和藤椒中天然含有蟾毒色胺，但暂无相关毒副作用的报道，因此，相关研究还有待进一步开展。

**图4:**
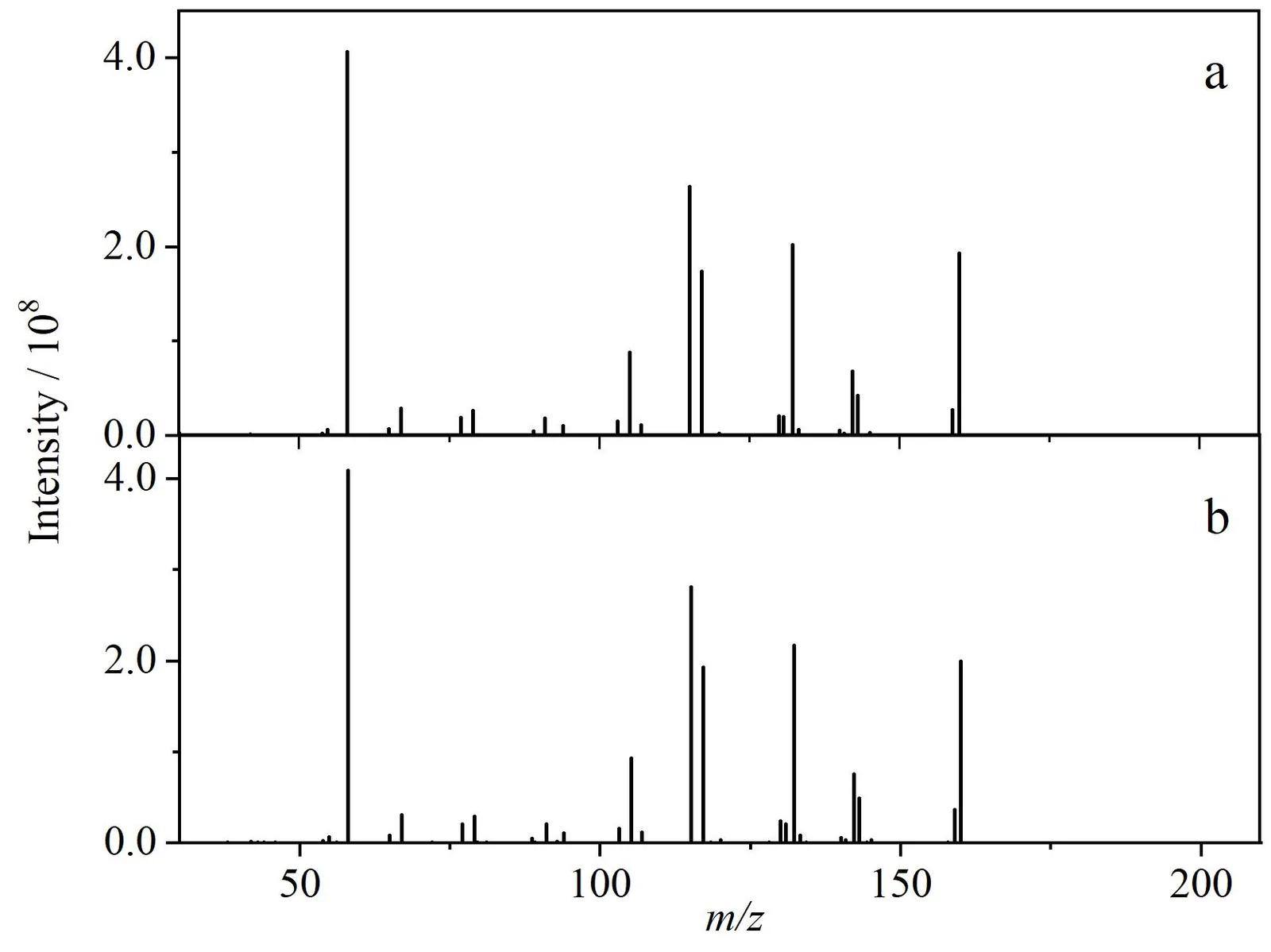
（a）蟾毒色胺标准品和（b）花椒样品的子离子扫描质谱图

**表6:** 花椒和藤椒样品中蟾毒色胺的检测结果（*n*=3）

Sample name	Bufotenine content/（mg/kg）
*Zanthoxylum armatum* DC. 1	367.9
*Zanthoxylum Bungeanum* Maxim 1	530.6
*Zanthoxylum Bungeanum* Maxim 2	476.5
*Zanthoxylum armatum* DC. 2	239.6
*Zanthoxylum Bungeanum* Maxim 3 （Dried）	35.9
*Zanthoxylum armatum* DC. 3	435.2
*Zanthoxylum armatum* DC. 4	507.9
*Zanthoxylum Bungeanum* Maxim 4	405.2
*Zanthoxylum Bungeanum* Maxim 5 （Dried）	63.5
*Zanthoxylum Bungeanum* Maxim 6 （Dried）	23.8
*Zanthoxylum Bungeanum* Maxim 7	885.0
*Zanthoxylum Bungeanum* Maxim 8	453.0

## 3 结论

本文结合强阳离子固相萃取柱净化、同位素稀释内标法校正、亲水柱分离，建立了测定食品基质中赛洛新、蟾毒色胺和赛洛西宾的UPLC-MS/MS检测方法。该方法简单、快速，准确度和灵敏度较高。同时本研究中还发现花椒和藤椒中天然含有蟾毒色胺。本方法的建立不仅填补了食品中非法添加色胺类物质检测方法的空白，为色胺类化合物的本底后续研究奠定基础，也为我国开展食品基质中色胺类化合物的安全性评价、司法鉴定等提供有力的技术支撑。

## References

[1] Yang L， Xiang P， Dang Y H， et al . Chinese Journal of Forensic Sciences， 2021（2）：24

[2] Li N， He H， Li F， et al . Chinese Journal of Pharmacology and Toxicology， 2022， 36（5）： 378

[3] Dasgupta A . Adv Clin Chem， 2017， 78： 163 10.1016/bs.acc.2016.07.004 28057187

[4] Geiger H A， Wurst M G， Daniels R N . ACS Chem Neurosci， 2018， 9（10）： 2438 10.1021/acschemneuro.8b00186 29956917

[5] Qian Z H， Hua Z D . Journal of Chinese Mass Spectrometry Society， 2021， 42（3）： 197

[6] Liu L Q， Chen J Z， Fu W S， et al . Chinese Journal of Chromatography， 2023， 41（11）： 976 10.3724/SP.J.1123.2023.07013 PMC10654875 37968816

[7] Sun Y， Su R B . Chinese Journal of Pharmacology and Toxicology， 2021， 35（4）： 241

[8] Malaca S， Faro A F L， Tamborra A， et al . Int J Mol Sci， 2020， 21： 9279 10.3390/ijms21239279 PMC7730282 33291798

[9] Xu X M， Du H， Yi H， et al . Chinese Traditional and Herbal Drugs， 2022， 53（23）： 7524

[10] European Monitoring Centre for Drugs and Drug Addiction . European Drug Report： Trends and Developments. （2021-06-09） ［2025-05-08］. https://www.emcdda.europa.eu/system/files/publications/13838/TDAT21001ENN.pdf https://www.emcdda.europa.eu/system/files/publications/13838/TDAT21001ENN.pdf

[11] Castellanos J P， Woolley C， Bruno K A . Reg Anesth Pain Med， 2020， 45（7）： 486 10.1136/rapm-2020-101273 32371500

[12] Johnson S， Black Q C . J Psychoactive Drugs， 2020， 52（4）： 319 10.1080/02791072.2020.1762023 32375602

[13] Carhart-Harris R L， Bolstridge M， Day C M J， et al . Psychopharmacology， 2018， 235（2）： 399 10.1007/s00213-017-4771-x PMC5813086 29119217

[14] Winkelman M . Curr Drug Abuse Rev， 2014， 7（2）： 101 10.2174/1874473708666150107120011 25563446

[15] Galvao-Coelho N L， Marx W， Gonzalez M， et al . Psychopharmacology， 2021， 238（2）： 341 10.1007/s00213-020-05719-1 PMC7826317 33427944

[16] Nutt D . Dialogues Clin Neurosci， 2019， 21（2）： 139 10.31887/DCNS.2019.21.2/dnutt PMC6787540 31636488

[17] Butler M， Seynaeve M， Nicholson T R， et al . Ther Adv Psychopharmacol， 2020， 10： 1 10.1177/2045125320912125 PMC7225815 32435447

[18] United Nations Office on Drugs and Crime . World Drug Report 2024. （2024-06） ［2025-05-12］. https://www.unodc.org/documents/data-and-analysis/WDR_2024/WDR24_Contemporary_issues.pdf https://www.unodc.org/documents/data-and-analysis/WDR_2024/WDR24_Contemporary_issues.pdf

[19] Yang Q， Cui M W， Cao Y， et al . Chinese Journal of Forensic Medicine， 2023， 38（3）： 339

[20] Alb ers C， Köhler H， Lehr M， et al . Int J Legal Med， 2004，118（6）： 326 10.1007/s00414-004-0469-9 15526212

[21] Honyiglo E， Franchi A， Cartiser N， et al . J Forensic Sci， 2019， 64（4）： 1266 10.1111/1556-4029.13982 30548541

[22] Bambauer T P， Wagmann L， Maurer H H， et al . Talanta， 2020（213）： 120847 10.1016/j.talanta.2020.120847 32200933

[23] Keller T， Keller A， Tutsch-Bauer E， et al . Forensic Sci Int， 2006， 161（2/3）： 130 10.1016/j.forsciint.2006.03.032 16831529

[24] Martin R， Schürenkamp J， Gasse A， et al . J Anal Toxicol， 2015， 39（2）： 126 10.1093/jat/bku141 25540060

[25] Martin R， Schürenkamp J， Gasse A， et al . Int J Legal Med， 2013， 127（3）： 593 10.1007/s00414-012-0796-1 23183899

[26] Björnstad K， Beck O， Helander A . J Chromatogr B， 2009， 877（11/12）： 1162 10.1016/j.jchromb.2009.03.004 19332394

[27] Gambaro V， Roda G， Visconti G L， et al . J Pharmaceut Biomed， 2016（125）： 427 10.1016/j.jpba.2016.03.043 27021629

[28] Sun W F， Xu J， Zhu F， et al . Chinese Journal of Food Hygiene， 2024， 36（12）： 1318

[29] Martin R， Schürenkamp J， Pfeiffer H， et al . Forensic Sci Int， 2014（237）： 1 10.1016/j.forsciint.2014.01.006 24513688

[30] Chen L， Huang X J . J Agric Food Chem， 2016， 64： 8684 10.1021/acs.jafc.6b03965 27787985

[31] Su X W， Chen Q J， Li R， et al . Journal of Hainan Medical University， 2025， 31（6）： 463

